# Reproducibility of small animal cine and scar cardiac magnetic resonance imaging using a clinical 3.0 tesla system

**DOI:** 10.1186/1471-2342-13-44

**Published:** 2013-12-17

**Authors:** Robert Manka, Cosima Jahnke, Thomas Hucko, Thore Dietrich, Rolf Gebker, Bernhard Schnackenburg, Kristof Graf, Ingo Paetsch

**Affiliations:** 1 Department of Cardiology, University Hospital Zurich, Rämistrasse 100, 8091, Zurich, Switzerland; 2 Institute for Biomedical Engineering, University and ETH Zurich, Zurich, Switzerland; 3 Institute of Diagnostic and Interventional Radiology, University Hospital Zurich, Zurich, Switzerland; 4 Department of Cardiology, University Hospital RWTH Aachen, Aachen, Germany; 5 Department of Cardiology, German Heart Institute Berlin, Berlin, Germany; 6 Philips Healthcare, Hamburg, Germany

## Abstract

**Background:**

To evaluate the inter-study, inter-reader and intra-reader reproducibility of cardiac cine and scar imaging in rats using a clinical 3.0 Tesla magnetic resonance (MR) system.

**Methods:**

Thirty-three adult rats (Sprague–Dawley) were imaged 24 hours after surgical occlusion of the left anterior descending coronary artery using a 3.0 Tesla clinical MR scanner (Philips Healthcare, Best, The Netherlands) equipped with a dedicated 70 mm solenoid receive-only coil. Left-ventricular (LV) volumes, mass, ejection fraction and amount of myocardial scar tissue were measured. Intra-and inter-observer reproducibility was assessed in all animals. In addition, repeat MR exams were performed in 6 randomly chosen rats within 24 hours to assess inter-study reproducibility.

**Results:**

The MR imaging protocol was successfully completed in 32 (97%) animals. Bland-Altman analysis demonstrated high intra-reader reproducibility (mean bias%: LV end-diastolic volume (LVEDV), -1.7%; LV end-systolic volume (LVESV), -2.2%; LV ejection fraction (LVEF), 1.0%; LV mass, -2.7%; and scar mass, -1.2%) and high inter-reader reproducibility (mean bias%: LVEDV, 3.3%; LVESV, 6.2%; LVEF, -4.8%; LV mass, -1.9%; and scar mass, -1.8%). In addition, a high inter-study reproducibility was found (mean bias%: LVEDV, 0.1%; LVESV, -1.8%; LVEF, 1.0%; LV mass, -4.6%; and scar mass, -6.2%).

**Conclusions:**

Cardiac MR imaging of rats yielded highly reproducible measurements of cardiac volumes/function and myocardial infarct size on a clinical 3.0 Tesla MR scanner system. Consequently, more widely available high field clinical MR scanners can be employed for small animal imaging of the heart e.g. when aiming at serial assessments during therapeutic intervention studies.

## Background

The accurate and reproducible assessment of cardiac function by measurement of leftventricular (LV) volumes, ejection fraction, and the amount of myocardial scar tissue is fundamental for evaluating heart diseases in cardiovascular research. A great advantage of cardiac magnetic resonance (CMR) imaging is the inherently high soft tissue contrast, and the possibility to image cardiac and vascular anatomy in a three-dimensional manner. The accuracy of CMR imaging for the evaluation of LV volumes, mass and scar amount has been validated in several human and small animal studies [1-6]. However, most animal studies have been conducted on dedicated ultra-high field small animal MR systems which are not widely available [6-8]. Thus, cardiac imaging in rodents using conventional clinical MR scanners would be highly desirable but remains technically demanding since imaging at high heart rates (> 300/min) will inadvertently limit the required high spatial and temporal resolution [9]. Recently, clinical MR scanners have been successfully employed to overcome these difficulties [10-12]. Cardiovascular research generally relies on reliable and accurate imaging protocols and such imaging protocols are mandatory for small animal examinations either; in particular the exact determination of LV volumes/mass and myocardial infarct size plays a pivotal role in therapeutic intervention studies [13]. In order to facilitate accurate and reproducible cardiac small animal imaging on commonly available clinical MR scanners, the present study determined inter-study, inter-reader and intra-reader reproducibility for the evaluation of LV volumes, mass and myocardial scar size in rats using a clinical 3.0 Tesla MR system.

## Methods

### Animal preparation

The study was approved by the Charité Institutional Review Board. Thirty-three adult Sprague–Dawley rats (7–16 weeks old) with a mean weight of 355.5 ± 91.8 g (range 269–589 g) were included in our study protocol. For surgical intervention, the animals were anesthetized with 0.3 ml Domitor® (1 mg/1 mL) + 0.8 ml Dormicum® (5 mg/1 mL)/kg BW. After thoracotomy, the left anterior descending coronary artery (LAD) was occluded on the level of the first diagonal branch by placing a snare ligature around the artery and a small portion of the surrounding myocardium. All animals underwent CMR imaging within 24 hours after surgery.

### CMR imaging

**Figure 1:**
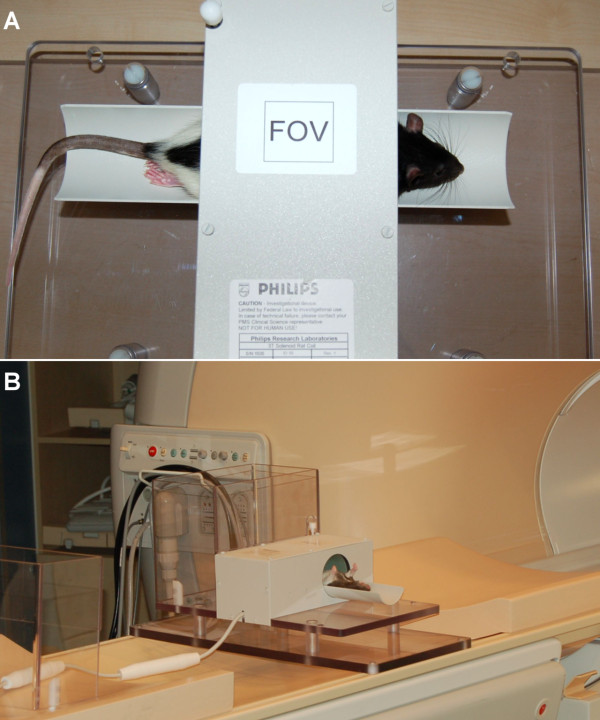
**Experimental Setup for CMR imaging of small animals (rat).** A specifically designed solenoid coil **(A)**, Philips Research Laboratory, Hamburg, Germany) with a diameter of 70 mm was placed perpendicular to the B_0_ field of the MR scanner **(B)** and favourably provided a high signal-to-noise ratio.

CMR imaging was performed using a clinical 3.0 Tesla MR system (Philips Healthcare, Best, The Netherlands) equipped with a Quasar Dual gradient system (40 mT/m, slew rate 200 T/m/sec) using a dedicated 70 mm solenoid receive-only coil placed perpendicular to B0 providing a high signal-to-noise ratio (Philips Research Laboratory, Hamburg, Germany). Anaesthesia was induced and maintained during the CMR examination with 3% isoflurane administered by a nasal cone. The animals were placed in a specially-designed cradle with four neonatal ECG electrodes (3 M™ Red Dot™ 2269 T) being attached to the paws in order to record an electrocardiogram for triggering of image data acquisition (see Figure 1). A dedicated software patch (GyroTools Ltd., Zurich Switzerland) installed on the scanner console enabled triggered image data acquisition up to heart rates of 400 beats per minute which constituted the only modification applied to the commercially available standard software of the scanner system. A multi-stack, multi-slice, segmented, spoiled gradient echo (GRE) pulse sequence was performed for localization of the heart in the three standard planes (transverse, sagittal, and coronal). For the assessment of LV volumes/mass whole heart coverage with contiguous short-axis cine imaging (8–10 slices) was carried out followed by acquisition of standard 4-and 2-chamber views.

For cine imaging a segmented gradient echo sequence was applied (turbo field echo; repetition time/echo time, 9.0 ms/4.2 ms; flip angle, 15°; temporal resolution 9.9 ms; in-plane spatial resolution, 0.4 x 0.4 mm^2^; slice thickness, 1.5 mm; field-of-view, 64 mm^2^; matrix, 159; total scan time approximately 20 minutes). Following cine imaging, a bolus of gadolinium-DTPA (dosage, 0.5 mmol/100 g BW) was administered via a catheter placed in a tail vein. After a 15 minute waiting period, full left-ventricular coverage, three-dimensional late enhancement (LE) imaging was performed in identical short axis geometry using an inversion prepared segmented gradient-echo-sequence (data acquisition window, 32 ms; time between 180° pulses = 2 heart beats; echo time, 3.3 ms; flip angle, 15°; in-plane spatial resolution, 0.3 x 0.3 mm^2^; slice thickness, 1.6 mm; field-of-view, 55 mm^2^; matrix, 188; total scan time approximately 14 minutes). The delay of the inversion prepulse was determined from an inversion prepared cine scan (Look-Locker sequence) and individually adjusted to optimally suppress signal from normal myocardium. All acquisitions were performed during free breathing with respiratory motion being partially compensated for by employing signal averaging (Number of Signal Averages, NSA = 5 for cine imaging and NSA = 2 for LE imaging).

### Image analysis

All data sets were evaluated using a standard analysis software (ViewForum Release 5.1, Philips Healthcare, Best, The Netherlands).

#### Assessment of LV volumes

**Figure 2:**
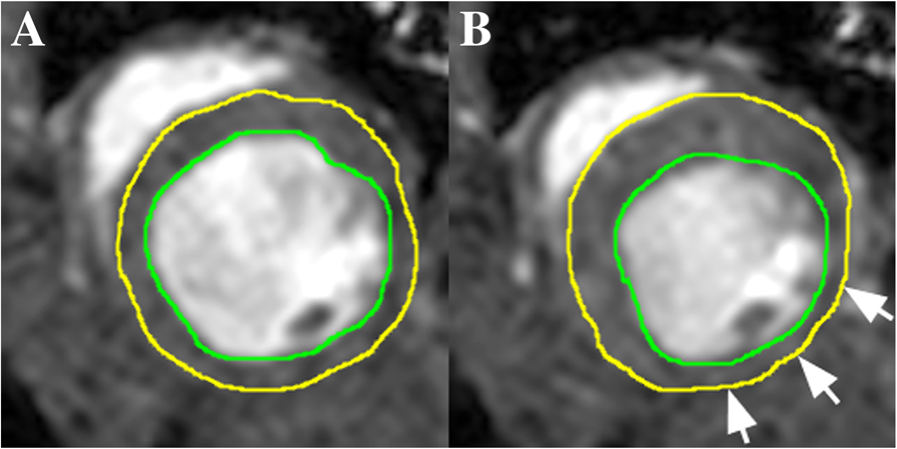
**Representative imaging example of endo-and epicardial contour delineation.** LV endocardial contours of the heart were traced manually at end-diastole **(A)** and end-systole **(B)** to calculate LV volumes according to Simpson’s rule. In addition, LV epicardial contours were traced manually to calculate LV mass; papillary muscles were excluded. White arrows indicate akinetic myocardial segments due to myocardial infarction.

Endocardial borders were traced manually at end-diastole and end-systole (Figure 2) with endocardial trabeculae being excluded; basal short axis slices demonstrating > 50% of visible circumferential LV myocardium were included in the analysis. LV end-diastolic volume (LVEDV) and LV end-systolic volume (LVESV) were calculated by applying Simpson’s rule with LV ejection fraction (LVEF) derived accordingly. In addition, end-diastolic epicardial borders were traced manually for measurement of end-diastolic LV mass (specific myocardial density, 1.05 g/cm3). LV end-diastolic diameter (LVEDD) was measured in a basal short-axis view according to standard definitions.

#### Assessment of LV scar amount

**Figure 3:**
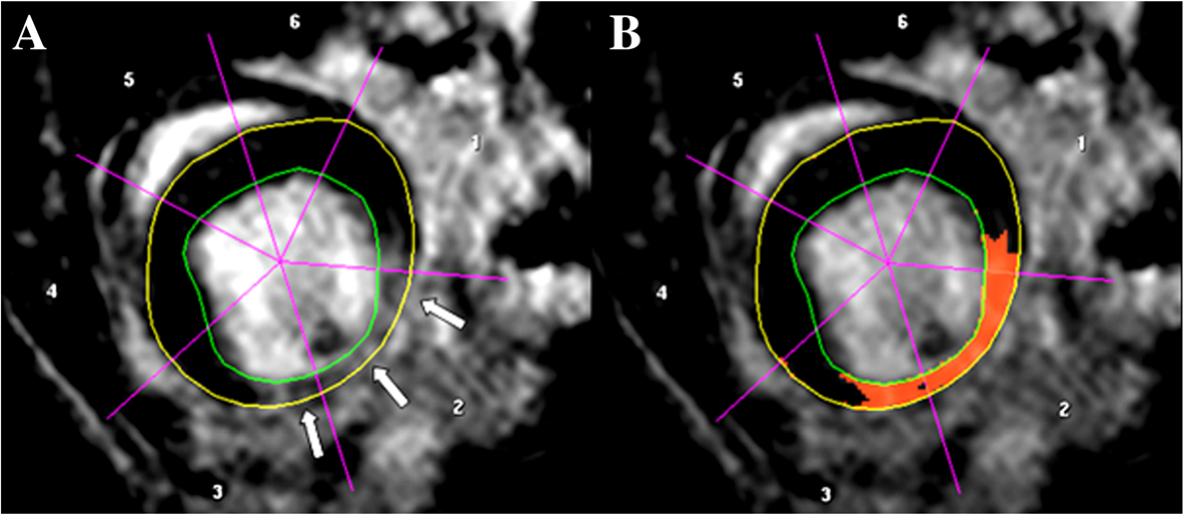
**Representative imaging example of endo-and epicardial contour delineation for the assessment of myocardial scar amount.** White arrows indicate infarcted region **(A)**. Red-coloured overlay represents segmented myocardial scar tissue **(B)**.

Endo-and epicardial borders were manually traced on LE images. The amount of scar was quantified by manual segmentation with the signal intensity threshold set to >2 standard deviations above the mean signal intensity of a remote myocardial region (Figure 3). The volume of enhanced myocardium was determined according to the disc summation method and provided in microliter (μl); in addition percentage of scar of total LV mass was calculated.

#### Reproducibility

The intra-reader and inter-reader reproducibility of LV volumes and mass were evaluated in all animals. To assess intra-reader reproducibility, left-ventricular contours were redrawn by the original observer (T.H., >4 years experience in reading >500 CMR studies per year) one month after the initial analysis. In order to assess inter-reader reproducibility an additional observer (R.M., >7 years experience in reading >600 CMR studies per year) carried out a separate analysis. For the assessment of inter-study reproducibility, 6 randomly chosen animals were scanned twice within 24 hours and evaluated by the first observer being blinded to the animal data.

### Statistical analysis

Statistical analyses were performed using SPSS, version 17.0, software (SPSS, Chicago). Values are reported as means ± standard deviations. The mean bias between the reads and their limits of agreement were determined according to the method described by Bland and Altman [14] and by calculating Lin's concordance coefficient [15] using MedCalc® Version 9.2.1.0 (MedCalc Software, Belgium). In addition, the coefficient of variability (CV) was derived [16]. All tests were two sided, and P < 0.05 was considered to indicate statistical significance.

## Results

### Study population

**Figure 4:**
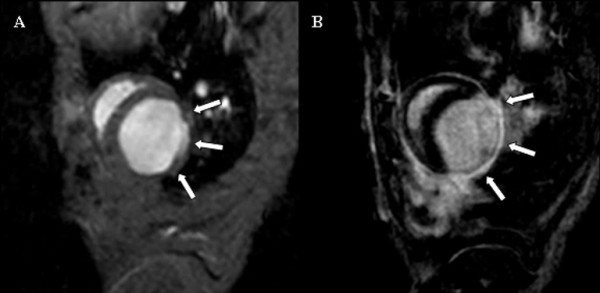
**Comparison of cine and late enhancement imaging (identical slice geometry).** Representative example showing an end-diastolic cine short-axis frame **(A)** and the corresponding late enhancement image **(B)**. White arrows indicate the akinetic **(A)** and infarcted region **(B)**; see also movie 1 (see additional file 1).

Image data acquisition was successfully completed in 32 out of 33 animals (97%) and in all 6 animals (100%) undergoing repeat CMR examinations. Heart rate ranged from 270 to 310 beats per minute (bpm), one CMR examination suffered from ECG-related triggering problems and could not be finalized. All 32 completed CMR examinations were of diagnostic image quality and constituted the final study population for analysis (for a representative imaging example see Figure 4 and Additional file 1).

### Left ventricular measurements

LVEF, LVEDV and LVEDD ranged from 23.0 to 68.8%, 257.0 to 609.2 μl and 5.3 to 8.7 mm, respectively; LV mass demonstrated values between 199.4 and 450.4 mg and myocardial scar volume ranged from 0 to 99.9 μl. The absolute values of all parameters are summarized in Table 1.

**Table 1:** Intra- and inter-reader reproducibility in 32 rats

**Intra-reader reproducibility of left ventricular measurements in 32 rats**							
	**Read 1 [mean ± SD]**	**Read 2 [mean ± SD]**	**% Bias**	**[95% - CI]**	**Lins’s, *Pc***	**[95% - CI]**	**CV**
**LVEDV [μl]**	453.0 ± 96.6	464.0 ± 113.8	-1.7	[-17.3; 14.0]	0.92	[0.84; 0.96]	0.09
**LVESV [μl]**	244.4 ± 105.8	250.5 ± 109.3	-2.2	[-25.7; 21.3]	0.97	[0.94; 0.99]	0.1
**LVEF [%]**	47.9 ± 14.9	47.6 ± 15.2	1.0	[-17.6; 19.5]	0.96	[0.91; 0.98]	0.09
**LVEDD [mm]**	7.6 ± 0.9	7.8 ± 1.0	-3.3	[-20.2; 13.6]	0.72	[0.52; 0.85]	0.09
**LV mass [mg]**	306.4 ± 54.8	315.0 ± 57.2	-2.7	[-23.7; 18.2]	0.82	[0.67; 0.91]	0.1
**LV scar [μl LV mass]**	41.8 ± 27.6	43.2 ± 28.2	-1.2	[-36.9; 34.6]	0.97	[0.94; 0.99]	0.15
**LV scar [% LV mass]**	14.4 ± 9.4	14.3 ± 9.5	2.1	[29.1; 33.4]	0.97	[0.94; 0.99]	0.15
**Inter-reader reproducibility of left ventricular measurements in 32 rats**							
	**Reader 1 [mean ± SD]**	**Reader 2 [mean ± SD]**	**% Bias**	**[95% - CI]**	**Lins’s, *Pc***	**[95% - CI]**	**CV**
**LVEDV [μl]**	453.0 ± 96.6	440.4 ± 105.1	3.3	[-23.5; 30.1]	0.82	[0.66; 0.91]	0.13
**LVESV [μl]**	244.4 ± 105.8	234.1 ± 108.6	6.2	[-24.0; 36.4]	0.93	[0.89; 0.97]	0.14
**LVEF [%]**	47.9 ± 14.9	49.5 ± 13.6	-4.8	[-31.6; 21.9]	0.93	[0.87; 0.96]	011.
**LVEDD [mm]**	7.6 ± 0.9	7.8 ± 1.0	-3.5	[-17.5; 10.5]	0.81	[0.65; 0.90]	0.07
**LV mass [mg]**	306.4 ± 54.8	313.9 ± 64.0	-1.9	[-50.1; 45.7]	0.83	[0.68; 0.91]	0.11
**LV scar [μl LV mass]**	41.8 ± 27.6	43.3 ± 28.7	-1.8	[-35.7; 32.1]	0.94	[0.89; 0.97]	0.22
**LV scar [% LV mass]**	14.4 ± 9.4	14.1 ± 9.5	3.8	[-31.7; 39.2]	0.93	[0.86; 0.97]	0.25

### Intra- and inter-reader reproducibility

**Figure 5:**
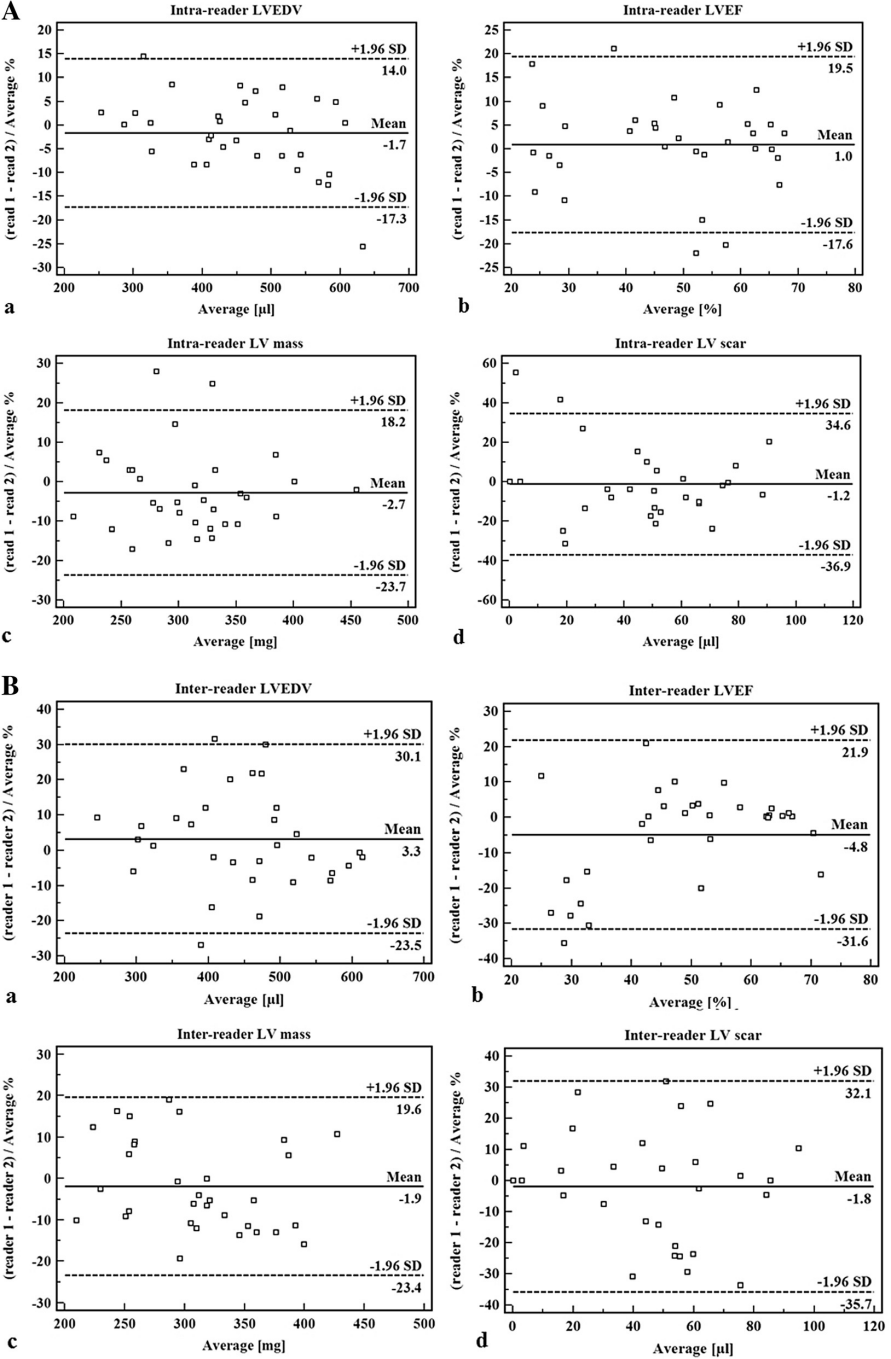
**Bland-Altman plots.** Intra-reader **(A)** and inter-reader **(B)** agreement for the assessment of **(a)** left ventricular end-diastolic volume, **(b)** left ventricular ejection fraction, **(c)** left ventricular mass, and **(d)** left ventricular scar volume, respectively.

Bland-Altman plots of intra-and inter-reader agreement are shown in Figure 5A and B, respectively. Calculation of Lin’s concordance correlation coefficient confirmed a substantial intra-reader agreement with regard to LV volume assessment and myocardial scar amount (range, 0.92 to 0.97); inter-reader agreement for the assessment of LV volumes and scar amount was moderate (range, 0.82 to 0.95, see Table 1).

### Inter-study reproducibility

The bias of repeat CMR examinations was found to be minimal for all left-ventricular parameters (Table 2). In addition, calculation of Lin’s concordance correlation coefficient resulted in a substantial inter-study agreement for the assessment of LV volumes and scar amount (range, 0.84 to 0.96, see Table 2).

**Table 2:** Inter-study reproducibility assessed in 6 randomly chosen rats

**Inter-study reproducibility of left ventricular measurements in 6 rats**							
	**Study 1 [mean ± SD]**	**Study 2 [mean ± SD]**	**% Bias**	**[95% - CI]**	**Lin’s *Pc***	**[95% - CI]**	**CV**
**LVEDV [μ]**	524.9 ± 29.4	525.1 ± 39.7	0.1	[-7.6; 7.8]	0.84	[0.39; 0.97]	0.04
**LVESV[μ]**	296.5 ± 74.3	302.7 ± 79.0	-1.8	[-14.0; 10.4]	0.96	[0.79; 0.99]	0.06
**LVEF [%]**	43.6 ± 13.8	42.8 ± 12.6	1.0	[-20.8; 22.9]	0.95	[0.71; 0.99]	0.1
**LVEDD [mm]**	8.4 ± 0.5	8.5 ± 0.5	-1.0	[-7.2; 5.2]	0.85	[0.29; 0.98]	0.03
**LV mass [mg]**	361.7 ± 127.5	379.8 ± 128.6	-4.6	[-38.2; 29.0]	0.89	[0.42; 0.98]	0.16
**LV scar [μl LV mass]**	66.6 ± 44.1	69.6 ± 39.6	-6.2	[-43.3; 31.0]	0.94	[0.64; 0.99]	0.22
**LV scar [% LV mass]**	19.2 ± 8.1	19.5 ± 8.7	-1.6	[-24.2; 21.0]	0.95	[0.72; 0.99]	0.13

## Discussion

The present study validated the use of a clinical 3.0 Tesla MR system for cardiac cine and scar imaging in small animals. Primarily, small rodent imaging is performed on dedicated animal MR systems with field strengths ≥ 4.7 Tesla with gradient and coil systems especially developed for small animal imaging. However, these MR scanner systems are not widely available and beyond that, require expensive investments. To overcome these restrictions, clinical MR systems may have the potential to represent a valuable alternative for accurate assessment of cardiovascular anatomy and function in small rodents. However, several key items need to be considered when performing CMR examinations in small animals: First, robust ECG monitoring is essential to ensure reliably triggered image data acquisition. Second, anaesthesia and animal temperature have to be monitored and maintained throughout the complete CMR examination in particular when aiming at serial examinations. Finally, in order to achieve an optimal signal-to-noise ratio resulting in high quality CMR images, high magnetic field strengths need to be combined with dedicated small animal radiofrequency receiver coils. In the present study, a clinically employed 3.0 Tesla system was introduced for the purpose of small animal imaging. In order to increase image SNR and minimize breathing artefacts, signal averaging was mandatory thereby accepting an increased total scan duration. In addition, for cine imaging a segmented gradient echo sequence was chosen over the commonly used steady-state free precession (SSFP) sequence type since the latter resulted in poor image quality mainly due to flow-/bloodpool-related artifacts with impairment of endocardial visibility (e.g. in the region of mitral inflow/aortic root). However, small animal CMR imaging at 3.0 Tesla proved to be feasible with a success rate of >97%. Recently, Saleh et al. [10] proved that long-term left ventricular remodelling in rat hearts after myocardial infarction can be rapidly and accurately assessed using a clinical 3.0 Tesla System with a dedicated small animal coil and a gradient echo sequence. In addition, quantitative assessment of cardiac volumes, mass and infarct size provided robust results with high inter-study, inter-reader and intra-reader reproducibility. The high reproducibility for the quantification of left-ventricular volumes and mass was in accordance to previously published data by Montet-Abou et al. [17] who investigated nine normal rats with four standard MR fast gradient- echo sequences using a 1.5 Tesla MR system. However, inter-, and intra-reader reproducibility was limited to four rats. Jones et al. [18] investigated 11 rats in an ischemia-reperfusion model and showed similar inter-study (3.3% vs. 4.8%) and intra-study (1.6% vs. 1.0%) reproducibility for the assessment of LVEF.

Accurate depiction of myocardial infarct size in animal models of cardiac diseases is crucial; hence, late enhancement imaging was incorporated in the current study protocol. Recently, Voelkl et al. [12] showed that widely available clinical 1.5 Tesla scanners enable high quality and accurate assessment of infarct size and functional parameters in mice. In our study quantitative analysis of myocardial infarct size demonstrated high inter-study, inter-reader and intra-reader reproducibility. The overall high reproducibility of quantitative CMR measurements may allow for the reliable and accurate assessment of even small treatment effects during serial examinations with the additional benefit of reducing sample size. In general, CMR imaging has been proposed the imaging method of choice to assess treatment effects in clinical and animal studies due to its high accuracy and low inter-study variability [6,16]. Based on the results of the present study conducted with the use of a conventional clinical 3.0 Tesla MR system a more widespread use of CMR imaging in small animal studies can be encouraged. In particular, the combined protocol of functional and morphological imaging of the rat heart is advantageous and can be integrated in pharmacological or interventional treatment studies in small animals.

### Limitations

Even though clinical MR scanners are more abundant than dedicated animal MR scanners, they may not always be available for animal studies and/or local rules may prevent their use. Image quality of small animal imaging on clinical scanners is generally restricted by limited shim capabilities which may be addressed by introducing high-order shimming. In addition, for small objects like rats and mice, the noise not only arises from physiological and thermal object noise but is rather dominated by noise from the electronic pathways; hence, a fully digital broadband system with analog to digital signal conversion in the radiofrequency coil may prove beneficial. Taking advantage of such technical improvements may allow for example to use steady-state free precession imaging sequences.

## Conclusions

The present study showed that a conventional clinical 3.0 Tesla MR scanner environment can be adapted to achieve high quality functional and morphological cardiac MR imaging of rats in vivo. Measurements of LV functional parameters and scar amount were highly reproducible. Consequently, the performance of therapeutic interventions aiming at the improvement of contractile function and the reduction of myocardial scar amount (e.g. stem cell therapy) may be studied in small animal models using widely available clinical CMR scanners.

## Competing interests

The authors declare that they have no competing interests.

## Authors’ contributions

RM, CJ, IP, BS and KG conceived of the study, participated in its design and coordination and performed the statistical analysis. RM, CJ, RG and IP carried out the imaging studies and drafted the manuscript. RM and TH carried out image analysis. TH and TD carried out animal preparation and experimental setup. All authors read and approved the final manuscript.

## Pre-publication history

The pre-publication history for this paper can be accessed here:

http://www.biomedcentral.com/1471-2342/13/44/prepub

## Supplementary Material

Additional files 1 **Comparison of cine and late enhancement imaging.** Cine and late enhancement imaging revealed akinetic wall motion and corresponding myocardial scar tissue resulting from myocardial infarction (same animal as in Figure 4). Click here for file
